# The BC Generations Project as a Tumor Tissue Resource for Cancer Research

**DOI:** 10.3390/curroncol29020107

**Published:** 2022-02-19

**Authors:** Umaimah Zanif, Jessica Chu, Jonathan Simkin, Trevor Dummer, Ryan Woods, Eric Belanger, Parveen Bhatti

**Affiliations:** 1 Cancer Control Research, BC Cancer Research Institute, Vancouver, BC V5Z 1L3, Canada; uzanif@bccrc.ca (U.Z.); jchu@bccrc.a (J.C.); jonathan.simkin@bccancer.bc.ca (J.S.); rwoods@bccancer.bc.ca (R.W.); 2 School of Population and Public Health, Faculty of Medicine, University of British Columbia, Vancouver, BC V6T 1Z4, Canada; trevor.dummer@ubc.ca; 3 Faculty of Health Sciences, Simon Fraser University, Burnaby, BC V5A 1S6, Canada; 4 Department of Pathology and Laboratory Medicine, Faculty of Medicine, University of British Columbia, Vancouver, BC V6T 1Z4, Canada; eric.belanger@vch.ca

**Keywords:** cohort study, tumor tissue, tumor repository

## Abstract

Population-based cohort studies can be a resource for tumor specimens, annotated with demographic, lifestyle, and health history data, that support innovative studies of cancer. Our aim was to establish and test a process for accessing tumor samples, held at pathology laboratories around British Columbia (BC), for participants of the BC Generations Project (BCGP). Through the BC Cancer Registry, we identified pathology reports for 1100 (93%) of the 1180 incident solid cancer cases diagnosed in BCGP as of 2019. Using manually abstracted data from the reports, we successfully retrieved 183 (92%) of the 200 formalin-fixed, paraffin-embedded (FFPE) blocks (breast, lung, bladder, and pancreas cancer cases) that we requested from pathology laboratories. No important differences in retrieval rates by cancer site, sample location (Greater Vancouver vs. Outside Greater Vancouver), sample type (biopsy vs. excision) or year of diagnosis were identified. A text mining solution recently implemented by the Registry will allow us to automate the process for data abstraction and should capture pathology reports for 100% of all newly diagnosed BCGP cancer cases moving forward. This will further enhance the utility of BCGP as a high-quality tumor tissue research resource.

## 1. Introduction

Cancers of the same anatomical site can be heterogeneous diseases with distinct molecular characteristics that predict prognosis and response to treatment. There is growing recognition that such cancers may also have distinct risk factors [1]. For example, microsatellite instability (MSI) in colorectal tumors is a strong prognostic factor for patient survival [2], and associations between BMI and colorectal cancer risk have been shown to significantly differ by tumor MSI status [3].

Researchers are increasingly interested in accessing tumor samples to identify distinct molecular subtypes and evaluate subtype-specific disease etiology. To support epidemiologic studies, these samples will need to come from well-characterized, prospectively followed study populations with detailed data on a variety of demographic, lifestyle, and environmental factors. These data are also increasingly important for clinical studies as they can be useful predictors of prognosis and treatment response [4,5].

Various large-scale cohort studies from around the world have established tumor tissue repositories to support innovative research [6,7,8,9]. Currently, there are no cohort studies in Canada with similarly available resources. For example, the Canadian Partnership for Tomorrow’s Health (CanPath), Canada’s largest cohort study, provides access to a wealth of data and biospecimens, specifically blood and urine samples, that can be evaluated in association with incidence of cancer for up to ~300,000 participants [10]. However, CanPath, and its regional cohorts, including the BC Generations Project (BCGP), lack the necessary resources to establish and maintain physical tumor biorepositories. Given that pathology departments across Canada are required to store tumor samples, long-term, after pathology review is completed (e.g., samples are stored as formalin fixed paraffin embedded (FFPE) blocks for at least 20 years by pathology laboratories in British Columbia (BC)), it may be possible to establish processes by which CanPath’s regional cohorts can facilitate access to tumor samples held at the pathology laboratories. In this manuscript, we describe such a process for accessing tumor samples for incident cancer cases among BCGP study participants, including testing of the process by attempting retrieval of a subset of samples.

## 2. Methods

### 2.1. Study Population

The BCGP, one of CanPath’s contributing regional cohorts [10], includes 29,850 participants, aged 35–69, that were recruited from across BC in 2009–2016 [11]. At baseline, participants completed a detailed health and lifestyle questionnaire, provided physical measurements (e.g., height and weight for calculation of BMI) and urine and venous blood samples. Participants have since completed additional follow-up questionnaires for updated health and lifestyle data.

### 2.2. Cancer Diagnoses

The BC Cancer Registry is a population-based registry that receives pathology reports from hospital and regional pathology services to ascertain primary cancer diagnoses. Annually, BCGP participants are linked to the BC Cancer Registry to identify incident cancers. The Registry does not record the data needed for the identification and retrieval of tumor specimens from the pathology reports. Instead, working with analysts in the BC Cancer Surveillance & Outcomes, Data and Analytics Group and the Provincial Health Services Authority (PHSA) Information Management/Information Technology Services (IMITS), we linked the first name, last name, date of birth, sex, and personal health number (PHN) of each incident BCGP solid cancer case to all pathology reports stored for that case within the Cancer Agency Information System (CAIS). Through this linkage, we obtained ~5000 documents, each one representing a pathology report or an addendum to a previous pathology report.

### 2.3. Tumor Pathology Database

Pathology reports linked with each BCGP cancer case were manually reviewed to identify those reports relevant to the cancer of interest by comparing cancer site and date of pathology review in the report to the cancer site and date of diagnosis provided through linkage with the Registry. We restricted review to solid cancers. Microsoft Access was used to create a database to house information abstracted from each of the pathology reports. For each case-specific report, the following information was entered into the database: (1) name and address of the pathology laboratory; (2) accession number; (3) report date; (4) sample type (i.e., biopsy or excision); (5) total number of FFPE blocks created; and (6) block IDs containing the tumor specimen.

### 2.4. Retrieving Tumor Samples

From among the successfully abstracted pathology reports with an identifiable sample location (within the Province of British Columbia) and sample type information, we randomly selected a total of 200 breast, lung, bladder, and pancreatic cancer cases for which to attempt the retrieval of samples from pathology laboratories. We selected these cancer sites as they capture a representative range of cancer characteristics (e.g., tumor sizes) with which to evaluate the effectiveness of our access process. Among those cases for whom both biopsy and resection samples were identified, the sample source was randomly selected.

Access to tumor samples was granted at the health authority level, including those within Greater Vancouver (Provincial Health Services Authority, Providence Health Care, Vancouver Coastal Health, and Fraser Health) and those outside Greater Vancouver (Vancouver Island Health Authority, Interior Health, and Northern Health). Though specific cost structures and procedures for accessing tumor samples did vary across the health authorities, in general, the access process required submission of a study protocol, Research Ethics Board certificate, list of accession numbers with block IDs and application forms specific to each health authority to a research coordinator. If the pathology report specified the FFPE blocks containing tumor tissue, we requested those blocks. If blocks containing the tumor were not specified, we requested all available blocks. Applications were typically reviewed by pathology laboratory personnel. Once the applications were approved, blocks were shipped directly to Dr. Eric Belanger, pathologist and study co-investigator, at Vancouver General Hospital for visual inspection. All samples were returned to the pathology laboratories within six months.

### 2.5. Data Analysis

Any solid cancer sites with less than 20 cases diagnosed in BCGP were grouped together as “Other”. This included cancers of the brain, liver, stomach, esophagus, cervix, larynx, and testes. We evaluated, by cancer site, the number and proportion of cases for which a pathology report was successfully identified. Because knowledge of the location is critical to sample retrieval, any pathology reports from which sample location could not be identified were considered missing. Among those cases for which pathology reports with sample location information was identified, we evaluated, stratified by cancer site, the number and proportion of cases by sample location (pathology lab located in Greater Vancouver area, pathology lab located Outside Greater Vancouver, or Both), sample type (Biopsy only, Excision only, Both, or Unknown), and year of cancer diagnosis (2009–2014 or 2015–2019). For sample location, “Both” captured those cases with multiple biopsy samples, multiple excision samples or both biopsy and excision samples, with at least one sample located in Greater Vancouver and at least one sample located outside of Greater Vancouver.

For the 200 cases that we requested samples, we examined, stratified by cancer site, the number and proportion of samples returned by sample location, sample type and diagnosis year. We also calculated an overall success rate for tumor sample acquisition by multiplying the proportion of pathology reports identified for breast, lung, bladder, and pancreas cancers by the proportion of samples for all these tumors that were successfully received.

## 3. Results

### 3.1. Tumor Pathology Database

As of December 31, 2019, a total of 1180 incident solid cancers had been diagnosed among BCGP participants, with the majority being cancers of the breast (*n* = 384) and prostate (*n* = 210). A pathology report with sample location information was successfully identified for 1100 (93%) of these solid cancers. Pathology reports could not be identified for 75 cases, and an additional five cases had pathology reports with missing sample location information. The proportion of reports successfully identified did vary by cancer site. At least 85% of reports were identified across each of the individual cancer sites except for pancreatic cancer, for which only 65% of reports were identified (Table 1).

Breakdowns of the cancers for which reports were identified by sample location, type and diagnosis year are provided in Table 2. Overall, the bulk of identified reports were from pathology laboratories within the Greater Vancouver area (57%), though this did vary by cancer site; for bladder cancer, 46% were from pathology laboratories in Greater Vancouver, whereas for kidney cancer, 77% were from pathology laboratories in Greater Vancouver. Only five identified reports came from outside of the Province. Overall, both biopsy and excision reports were only identified for 387 of the 1100 cancers (35%). When looking at tumors for which at least a biopsy report was available, biopsy reports were identified for 897 of the 1100 cancers (82%). The sample type could not be identified for only 13 cases. The percentage of sample types did vary considerably by cancer site (Table 2). For example, excision only samples were identified for 82% of kidney cancers, whereas excision only samples were identified for just 4% of prostate cancers. The percentage of cancers for which pathology reports were identified did not vary considerably by year of diagnosis.

### 3.2. Tumor Tissue Samples

In 52 of the 200 pathology reports (26%), the blocks containing the tumor specimen were not specified, so all available blocks (up to 87) were requested. Requests to the seven different health authorities were all submitted by January 2021. Approvals were successfully obtained from all sites by February, and, by March 2021, samples had been received from all but one of the health authorities. Due to staffing shortages, samples from the remaining health authority were not received until September 2021.

We received 183 (92%) of the requested samples. The remaining 17 samples were out for other studies or could not be located by the pathology laboratory. There was some variation in retrieval rates by cancer site; only 82% of requested pancreas tumor samples were retrieved, whereas 96% of lung tumor samples were retrieved. Table 3 provides a breakdown of the requested and retrieved samples by sample location, sample type and diagnosis year. Overall, there was greater success in obtaining biopsy samples (95%) as compared to excision samples (87%). No overall differences in retrieval were observed by sample location or diagnosis year. For bladder cancer, greater success with retrieval occurred with samples from outside Greater Vancouver (96%) versus within Greater Vancouver (76%). Multiplying the overall proportion of pathology reports successfully identified for breast, lung, bladder, and pancreas cancers (95%) with the overall proportion of samples successfully retrieved (92%), we estimated an overall tumor sample acquisition rate of 87%.

## 4. Discussion

This exercise demonstrates the tremendous potential for BCGP to serve as a population-based resource for highly annotated tumor tissue samples. Of the 1180 incident solid cancers diagnosed within BCGP, we were only unable to identify pathology reports for 75 cases. This was attributed to a system malfunction that resulted in a loss of data within CAIS. However, it remains unclear as to why such an occurrence had a greater impact on pancreatic cancer pathology reports than the other cancer sites.

Regional differences in the numbers of identified reports were observed and likely reflected a combination of the population distribution of the BCGP, with most participants residing in the Greater Vancouver area [11], and regional differences in the incidence of specific cancers [12]. We identified relatively few cases with both biopsy and excision pathology reports. The availability of biopsy and excision samples for a particular cancer was mostly a function of the standard of care for that cancer. However, for a large proportion of cancers (82%), a biopsy report was identified. Having access to biopsy samples may be useful since any neoadjuvant therapies carried out before excision would impact tumor biology. However, tissue volumes associated with biopsy samples may be inadequate for certain research needs (e.g., tumor tissue microarray creation). BCGP is currently exploring opportunities to link with cancer treatment data for participants, allowing the identification of individuals who have undergone neoadjuvant treatment.

Ninety-two percent of requested samples were successfully retrieved. Twenty-six percent of the reports associated with these samples did not indicate which block specifically contained tumor tissue. For tumor samples associated with such reports to be useful for future research, an extra step will be required, where slides associated with the tumor blocks are first reviewed by a pathologist to identify the most relevant blocks. The 17 samples that we did not receive were primarily excision samples, and this is reflected in the lower retrieval rates seen for excision versus biopsy samples across the four cancer sites. The regional difference in the success of retrieval for bladder cancer is because the missing bladder tumor excision samples were mostly from the Greater Vancouver area.

Our overall tumor sample acquisition rate (87%) is higher than that reported by previous studies. For example, the Prostate, Lung, Colorectal and Ovarian Cancer (PLCO) Screening Trial only reported a sample retrieval success rate of 47% [7], with the Nurses’ Health Study reporting success rates of 70–80% [8].

The Province of BC has moved away from paper-based pathology reports to an electronic health record format called CareConnect. The BC Cancer Registry recently deployed the Electronic Mapping, Reporting, and Coding (eMaRC) system [13] to capture cancer-related pathology reports directed to CareConnect. Text mining is being used to extract necessary information from these reports to populate the Registry. We are currently working with the Registry to establish an automated text mining solution that would include data needed to support access of tumor samples. Not only would this allow us to identify 100% of all pathology reports for BCGP cases, but by eliminating the need for the manual abstraction of pathology reports, the efficiency of the BCGP tumor resource would be greatly enhanced.

Our evaluation of pathology report availability captured a comprehensive range of cancer sites, and our evaluation of tumor retrieval rates included four cancer sites that are broadly representative of incidence rates and tumor sizes of other solid cancers. Given the small number of cases for many of the cancer sites, we could not conduct detailed, site-specific evaluations of variation in report availability and tumor retrieval rates by sample location, sample type and diagnosis year.

Pending further development of the eMaRC automated data extraction platform, we believe that BCGP can serve as a high-quality resource for well-annotated tumor samples across a broad range of cancer sites. Cohort studies in other jurisdictions across Canada should explore the potential to establish similar resources.

## Figures and Tables

**Table 1:** Solid incident cancers, by site, diagnosed in BCGP (2009–2019) and proportion for which pathology reports were successfully identified.

Cancer Site	Cases Diagnosed in BCGP *N*	Pathology Reports Identified *N* (%)	
Breast	384	380	(99)
Prostate	210	195	(93)
Colorectal	115	102	(89)
Melanoma	90	78	(87)
Lung	84	75	(89)
Endometrial	73	72	(99)
Bladder	55	52	(95)
Other *	51	46	(90)
Pancreas	31	20	(65)
Kidney	26	22	(85)
Ovary	24	20	(85)
Oral	21	19	(91)
Thyroid	20	19	(95)
Total	1180	1100	(93)

* Includes cancers of the brain, liver, stomach, esophagus, cervix, larynx, and testes.

**Table 2:** Identified tumor pathology reports by sample location, type, and diagnosis year.

	Cases for Which Pathology Report was Identified (*n*, %)															
	Breast (*N* = 380)		Prostate (*N* = 195)		Colorectal (*N* = 102)		Melanoma (*N* = 78)		Lung (*N* = 75)		Endometrial (*N* = 72)		Bladder (*N* = 52)		Other (*N* = 46)	
Sample location																
Greater Vancouver	210	(55)	107	(55)	61	(60)	38	(49)	45	(60)	43	(60)	24	(46)	31	(67)
Outside Greater Vancouver	125	(33)	76	(39)	36	(35)	<40		30	(40)	20	(28)	28	(54)	<15	
Both	45	(12)	12	(6)	5	(5)	<5		0	0	9	(13)	0	0	<5	
Sample type																
Biopsy only	164	(43)	131	(67)	28	(27)	23	(31)	52	(69)	15	(21)	35	(67)	28	(61)
Excision only	26	(7)	8	(4)	24	(24)	30	(38)	12	(16)	17	(24)	11	(21)	8	(17)
Both	189	(50)	56	(29)	50	(49)	25	(32)	6	(8)	40	(56)	<10		10	(22)
Unknown	0	0	0	0	0	0	0	0	5	(7)	0	0	<5		0	0
Diagnosis Year																
2009–2014	179	(47)	96	(49)	62	(61)	46	(59)	40	(53)	34	(47)	30	(58)	24	(52)
2015–2019	201	(53)	99	(51)	40	(39)	32	(41)	35	(47)	38	(53)	22	(42)	22	(48)
	**Kidney** **(*N* = 22)**		**Pancreas** **(*N* = 20)**		**Ovary** **(*N* = 20)**		**Oral** **(*N* = 19)**		**Thyroid** **(*N* = 19)**		**Total** **(*N* = 1100)**					
Sample location																
Greater Vancouver	17	(77)	11	(55)	15	(75)	10	(53)	13	(68)	625	(57)				
Outside Greater Vancouver	5	(23)	<10		5	(25)	9	(47)	6	(32)	397	(36)				
Both	0	0	<5		0	0	0	0	0	0	78	(7)				
Sample type																
Biopsy only	<5		16	(80)	<5		12	(63)	<5		510	(46)				
Excision only	18	(82)	<5		16	(80)	<5		15	(79)	190	(17)				
Both	<5		<5		0	0	<5		<5		387	(35)				
Unknown	<5		<5		<5		<5		<5		13	(1)				
Diagnosis Year																
2009–2014	10	(45)	9	(45)	12	(60)	8	(42)	11	(58)	561	(51)				
2015–2019	12	(55)	11	(55)	8	(40)	11	(58)	8	(42)	539	(49)				

Any cells in the table with one to less than five observations were labelled as ‘<5’. In some instances, cells were labelled as ‘<10’, ‘<15’, or ‘<40’ to help obscure the low number of observations in adjacent cells.

**Table 3:** Tumor samples requested and received by sample location, type, and diagnosis year.

	Breast			Lung			Bladder			Pancreas			Total		
	Request *n* = 93	Receive *n* = 86 (92% *)		Request *n* = 45	Receive *n* = 43 (96% *)		Request *n* = 45	Receive *n* = 40 (89% *)		Request *n* = 17	Receive *n* = 14 (82% *)		Request *n* = 200	Receive *n* = 183 (92% *)	
Sample location															
Greater Vancouver	61	57	(93)	24	23	(96)	17	13	(76)	10	8	(80)	112	101	(90)
Outside Greater Vancouver	32	29	(91)	21	20	(95)	28	27	(96)	7	6	(86)	88	82	(93)
Sample type															
Biopsy	44	42	(95)	30	29	(97)	<40	<30		<15	<15		117	111	(95)
Excision	49	44	(90)	15	14	(93)	<20	<15		<5	<5		83	72	(87)
Diagnosis year															
2009–2014	45	42	(93)	24	23	(96)	27	25	(93)	9	7	(78)	105	97	(92)
2015–2019	48	44	(92)	21	20	(95)	18	15	(83)	8	7	(88)	95	86	(91)

* Percentage of those requested. Any cells in the table with one to less than five observations were labelled as ‘<5′. In some in stances, cells were labelled as ‘<15′, ‘<20′, ‘<30′, or ‘<40′ to help obscure the low number of observations in adjacent cells.

## Data Availability

The data presented in this study are available through a BC Generations Project data access application (https://www.bcgenerationsproject.ca/researchers/overview-of-data-and-samples/).

## References

[1] Hamada T., Keum N., Nishihara R., Ogino S. (2017). Molecular pathological epidemiology: New developing frontiers of big data science to study etiologies and pathogenesis. J. Gastroenterol..

[2] Kang S., Na Y., Joung S.Y., Lee S.I., Oh S.C., Min B.W. (2018). The significance of microsatellite instability in colorectal cancer after controlling for clinicopathological factors. Medicine.

[3] Campbell P.T., Jacobs E.T., Ulrich C.M., Figueiredo J.C., Poynter J.N., McLaughlin J.R., Haile R.W., Jacobs E.J., Newcomb P.A., Potter J.D. (2010). Case-control study of overweight, obesity, and colorectal cancer risk, overall and by tumor microsatellite instability status. J. Natl. Cancer Inst..

[4] Lugo D., Pulido A.L., Mihos C.G., Issa O., Cusnir M., Horvath S.A., Lin J., Santana O. (2019). The effects of physical activity on cancer prevention, treatment and prognosis: A review of the literature. Complement. Ther. Med..

[5] Peppone L.J., Mustian K.M., Morrow G.R., Dozier A.M., Ossip D.J., Janelsins M.C., Sprod L.K., McIntosh S. (2011). The Effect of Cigarette Smoking on Cancer Treatment–Related Side Effects. Oncologist.

[6] Milne R.L., Fletcher A.S., MacInnis R.J., Hodge A.M., Hopkins A.H., Bassett J.K., Bruinsma F.J., Lynch B.M., Dugué P.A., Jayasekara H. (2017). Cohort Profile: The Melbourne Collaborative Cohort Study (Health 2020). Int. J. Epidemiol..

[7] Zhu C.S., Huang W.-Y., Pinsky P.F., Berg C.D., Sherman M., Yu K.J., Carrick D.M., Black A., Hoover R., Lenz P. (2016). The Prostate, Lung, Colorectal and Ovarian Cancer (PLCO) Screening Trial Pathology Tissue Resource. Cancer Epidemiol. Prev. Biomark..

[8] Bao Y., Bertoia M.L., Lenart E.B., Stampfer M.J., Willett W.C., Speizer F.E., Chavarro J.E. (2016). Origin, Methods, and Evolution of the Three Nurses’ Health Studies. Am. J. Public Health.

[9] Zheng W., Chow W.-H., Yang G., Jin F., Rothman N., Blair A., Li H.-L., Wen W., Ji B.-T., Li Q. (2005). The Shanghai Women’s Health Study: Rationale, Study Design, and Baseline Characteristics. Am. J. Epidemiol..

[10] Dummer T.J.B., Awadalla P., Boileau C., Craig C., Fortier I., Goel V., Hicks J.M.T., Jacquemont S., Knoppers B.M., Le N. (2018). The Canadian Partnership for Tomorrow Project: A pan-Canadian platform for research on chronic disease prevention. CMAJ Can. Med. Assoc. J..

[11] Dhalla A., McDonald T.E., Gallagher R.P., Spinelli J.J., Brooks-Wilson A.R., Lee T.K., Lai C., Borugian M.J., Woods R.R., Le N.D. (2018). Cohort Profile: The British Columbia Generations Project (BCGP). Int. J. Epidemiol..

[12] BC Cancer Agency (2011). British Columbia 2011 Regional Cancer Report.

[13] eMaRC Plus|Registry Plus|CDC. https://www.cdc.gov/cancer/npcr/tools/registryplus/mp.htm.

